# Use of Epoetin Alfa for Anemia of Chronic Disease in a Jehovah’s Witness Teenager With Disseminated Coccidioidomycosis: A Case Report

**DOI:** 10.7759/cureus.102090

**Published:** 2026-01-22

**Authors:** Yoshihiro Ozaki, Danielle Herrmann

**Affiliations:** 1 Pediatric Hospital Medicine, Valley Children’s Hospital, Madera, USA; 2 Pharmacy, Valley Children's Hospital, Madera, USA

**Keywords:** anemia of chronic disease (acd), blood conservation, coccidioidomycosis, epoetin, erythropoiesis-stimulating agents, inpatient pediatrics, jehovah's witness

## Abstract

Anemia of chronic disease is a common complication of prolonged infection and systemic inflammation and can be difficult to manage in patients who decline blood transfusion, such as Jehovah’s Witnesses. Erythropoiesis-stimulating agents are widely used in renal and oncologic settings but are rarely reported in infection-associated anemia among pediatric or young adult patients. We describe a 19-year-old Jehovah’s Witness male with disseminated *Coccidioides immitis* infection complicated by severe anemia of chronic disease. Because blood transfusion was declined, epoetin alfa was administered as a transfusion-sparing strategy prior to surgical intervention. This case highlights a potential role of epoetin alfa as an alternative strategy for managing infection-related anemia in select patients when transfusion is not an option.

## Introduction

Anemia of chronic disease (ACD), also known as anemia of inflammation, is a frequent complication of prolonged infection and inflammatory states. It results from cytokine-mediated iron sequestration, suppression of erythropoietin production, and impaired erythroid proliferation. Erythropoiesis-stimulating agents (ESAs), such as epoetin alfa, have been used in renal and perioperative blood conservation settings [1-3]. However, their use in infection-associated ACD, particularly among pediatric or young adult Jehovah’s Witness patients, is rarely documented.

Coccidioidomycosis, caused by *Coccidioides immitis* and *Coccidioides posadasii*, is endemic to arid regions of the southwestern United States, with approximately 95% of U.S. cases reported from Arizona and California [4]. Within California, a substantial proportion of cases occur in the Central Valley, where environmental exposure is greatest. Kern County consistently reports the highest incidence rates statewide, and 2024 marked the highest annual number of reported cases on record according to the California Department of Public Health [5].

While most infections present as self-limited pulmonary disease, disseminated coccidioidomycosis affecting the bones, joints, or central nervous system occurs in approximately 1% of infected individuals [6] and can result in chronic systemic inflammation and secondary ACD. We present the case of a 19-year-old Jehovah’s Witness male from California’s Central Valley with disseminated *Coccidioides immitis* infection complicated by severe ACD, successfully treated with epoetin alfa in lieu of blood transfusion. This case underscores the potential role of ESAs in managing chronic infection-related anemia when transfusion is contraindicated or declined. The clinical dilemma in this case lies in managing severe infection-associated anemia requiring surgical intervention in a patient who declines transfusion, a scenario for which evidence guiding the use of ESAs remains limited.

## Case presentation

A 19-year-old previously healthy Jehovah’s Witness male from California’s Central Valley presented with several months of intermittent lower back and right shoulder pain, initially managed conservatively with analgesics. Over the following weeks, he developed fatigue, poor appetite, night sweats, and low-grade fevers accompanied by approximately 27 pounds of unintentional weight loss. He later noted progressive swelling over the right shoulder and upper back associated with worsening pain and limited range of motion.

He was evaluated at an outside hospital, where imaging revealed multiple abscesses and lytic bone lesions. Computed tomography demonstrated osteomyelitis of the scapular spinous process, lytic lesions involving the ribs, spine, pelvis, and proximal femur, as well as psoas, thoracic paraspinal, and abdominal wall abscesses. Empiric therapy with vancomycin, piperacillin-tazobactam, and fluconazole was initiated, and liposomal amphotericin B was later added for disseminated coccidioidomycosis. The Coccidioides complement fixation titer was 1:512. Magnetic resonance imaging of the cervical spine showed no evidence of discitis or osteomyelitis. Interventional radiology performed aspiration and drainage of a paraspinal abscess. The patient remained hospitalized at the outside facility for approximately three weeks, during which he received antifungal therapy and supportive care for pain control and nutrition.

The patient was subsequently transferred to our institution for inpatient rehabilitation. On arrival, he continued to experience significant right foot pain and was unable to bear weight on the right lower extremity. Given his non-weight-bearing status, prophylactic enoxaparin was initiated for deep vein thrombosis prevention. Magnetic resonance imaging of the right foot demonstrated osteomyelitis and septic arthritis involving the talonavicular and subtalar joints with adjacent fluid collections. Fluconazole at a dose of 800 mg daily was added to ongoing liposomal amphotericin B therapy. Laboratory evaluation (Table 1) revealed a hemoglobin level of 6.5 g/dL, mean corpuscular volume of 77.9 fL, red cell distribution width of 17.3%, serum iron of 14 µg/dL, transferrin of 124 mg/dL, and ferritin of 1,604 ng/mL, consistent with ACD. Baseline renal function was preserved, with a serum creatinine of 0.84 mg/dL prior to epoetin alfa initiation. Oral iron supplementation, which had been initiated at the outside hospital, was continued during hospitalization.

**Table 1:** Key Laboratory Findings Prior to Epoetin Alfa Administration MCV = mean corpuscular volume; RDW = red cell distribution width; BUN = blood urea nitrogen.

Laboratory Test	Reference Range	Results	Interpretation
WBC	3.7-11.2 x10^3^/µL	8.7	Normal
Platelet	238-378 x10^3^/µL	439	Mildly elevated
MCV	78.2-96.2 fL	77.9	Borderline low
RDW	11.8-15.5 %	17.3	Elevated
Reticulocyte	0.5-2.5 %	1.8	Inappropriately low for the degree of anemia
Serum iron	65-175 µg/dL	14	Low
Transferrin	180-382 mg/dL	124	Low
Transferrin saturation	20-50 %	9	Low
Ferritin	4.40-207.00 ng/mL	1,604	Markedly elevated
BUN	6.0-20.0 mg/dL	17	Normal
Creatinine	0.73-1.18 mg/dL	0.84	Normal

Orthopedic surgery was consulted and recommended irrigation and debridement of the right talonavicular and subtalar joints. Given the need for surgical intervention, options for anemia management were discussed with the patient and his family. Consistent with his religious beliefs as a Jehovah’s Witness, the patient declined blood transfusion except in life-threatening circumstances. Because he remained hemodynamically stable without evidence of severe symptomatic anemia, epoetin alfa (Epogen) was administered intravenously at a dose of 20,000 units (approximately 300 units/kg) once daily as a transfusion-sparing measure and was continued during the hospitalization. Over two days, the hemoglobin level increased from 6.5 g/dL to 7.3 g/dL. These laboratory findings supported the diagnosis of ACD, characterized by iron sequestration and suppressed erythropoiesis. The modest rise in hemoglobin, while limited, was clinically meaningful in permitting surgical intervention without transfusion in this individual case. The patient subsequently underwent irrigation and debridement without complication, and no transfusion was required. Cultures obtained from the operative specimen again grew *Coccidioides immitis*. Postoperative hemoglobin values remained stable at 6.5 g/dL on postoperative day one and increased to 7.5 g/dL by postoperative day five (Table 2).

**Table 2:** Hemoglobin and Hematocrit Trends Before and After Epoetin Alfa Administration POD = postoperative day

Laboratory Test	Reference Range	Pre-Epoetin	Post-Epoetin Day 2	POD #1	POD #5
Hemoglobin	12.3-17.1 g/dL	6.5	7.3	6.5	7.5
Hematocrit	36.4-49.5 %	19.6	22.3	19.3	22.1

Following surgical intervention, antifungal therapy with liposomal amphotericin B and oral fluconazole was continued under the guidance of infectious disease specialists. Over the subsequent four weeks, hemoglobin levels remained stable in the mid-7 g/dL range. The patient later developed right jaw pain, and magnetic resonance imaging revealed osteomyelitis of the mandibular condyle with adjacent soft tissue edema involving the right masseter muscle. Although complement fixation titers and inflammatory markers showed improvement, repeat imaging one month later demonstrated progressive disseminated disease with new and recurrent abscesses involving the jaw, lungs, scapula, ribs, spine, left hemipelvis, and right ankle. Following multidisciplinary discussion with infectious disease specialists, the patient was transferred to another institution for consideration of immunomodulatory therapy.

## Discussion

ACD, also referred to as anemia of inflammation, is a multifactorial condition driven by immune-mediated disturbances in iron metabolism and erythropoiesis. Proinflammatory cytokines, including interleukin-6 and tumor necrosis factor-α, stimulate hepatic hepcidin production, resulting in iron sequestration within macrophages and reduced intestinal iron absorption. These inflammatory mediators also suppress erythropoietin production and impair bone marrow responsiveness to erythropoietin, leading to a normocytic or mildly microcytic anemia characterized by low serum iron, low transferrin, and elevated ferritin levels [7]. This pathophysiologic pattern was evident in our patient, whose laboratory profile demonstrated severe anemia with low serum iron and transferrin, markedly elevated ferritin, and an inappropriately low reticulocyte response despite preserved renal function.

ESAs, such as epoetin alfa, are approved for the treatment of anemia associated with chronic kidney disease, myelosuppressive chemotherapy, and for reducing allogeneic blood transfusion requirements in elective surgical settings [3]. However, their use in infection-associated ACD, particularly among pediatric or young adult patients, remains poorly described. Most pediatric literature involving ESA therapy focuses on cardiac surgery or hemolytic uremic syndrome, where transient stimulation of erythropoiesis may reduce transfusion requirements. In chronic infectious or inflammatory conditions, available evidence is largely limited to small adult case series suggesting that ESAs may increase hemoglobin levels when endogenous erythropoietin production is insufficient to overcome inflammatory suppression [8,9].

Jehovah’s Witness patients present unique clinical challenges in the management of severe anemia due to their religious refusal of blood transfusions. This belief, rooted in biblical interpretation, precludes acceptance of whole blood and major blood components, necessitating alternative management strategies. In such cases, approaches including ESAs, iron supplementation, and meticulous perioperative blood conservation become essential. ESAs are generally considered ethically and clinically acceptable in this context, as they stimulate endogenous erythropoiesis without violating the doctrinal prohibitions of the Jehovah’s Witness faith [10]. A limited number of reports describe successful use of epoetin alfa in Jehovah’s Witness pediatric patients, including cases of hemolytic uremic syndrome and congenital heart disease, where transfusion was not an option [2,11].

This case illustrates a rare and ethically complex clinical scenario involving a Jehovah’s Witness adolescent with severe disseminated *Coccidioides immitis* infection complicated by profound ACD. The refusal of transfusion required the implementation of a transfusion-sparing strategy. Epoetin alfa was selected to augment erythropoiesis prior to surgical debridement and was associated with a modest but clinically meaningful increase in hemoglobin within two days, allowing surgery to proceed without transfusion in this individual case. This response suggests preserved marrow responsiveness once exogenous erythropoietin levels were restored, despite ongoing systemic inflammation.

The principal safety concerns associated with epoetin alfa include cardiovascular and thromboembolic complications, particularly in the setting of rapid hemoglobin increases. Reported adverse effects include hypertension, seizures, and thrombotic events [3]. In this case, the patient remained clinically stable, with no evidence of hypertension, seizure activity, or thromboembolism attributable to ESA therapy. Prophylactic enoxaparin was administered due to the patient’s non-weight-bearing status, which may have mitigated thromboembolic risk associated with immobilization and ESA use.

Disseminated coccidioidomycosis occurs in approximately 1% of infected individuals and frequently involves the bones, joints, or central nervous system, often resulting in prolonged systemic inflammation that exacerbates anemia [12]. To our knowledge, this is the first reported case describing the use of epoetin alfa for ACD secondary to disseminated fungal infection in a Jehovah’s Witness adolescent. This case underscores the importance of individualized, ethically sensitive, and multidisciplinary approaches to anemia management when conventional transfusion-based therapy is contraindicated or declined.

The patient and the patient’s family expressed understanding of the clinical course and supported publication of this case to contribute to medical education. Written informed consent for publication of this case report was obtained from the patient.

## Conclusions

This case demonstrates the successful use of epoetin alfa as a transfusion-sparing strategy in a Jehovah’s Witness adolescent with severe ACD secondary to disseminated coccidioidomycosis. In the setting of active infection and ongoing inflammation, epoetin alfa was associated with a timely and clinically meaningful increase in hemoglobin, allowing necessary surgical intervention to proceed without blood transfusion or observed adverse effects.

Although ESAs are not routinely used for infection-associated anemia in pediatric or young adult populations, this case highlights their potential role in carefully selected patients when transfusion is contraindicated or declined. The observed hematologic improvement represents a temporal association in a single patient with limited follow-up and concurrent interventions, and therefore should be considered hypothesis-generating rather than evidence of efficacy. Multidisciplinary collaboration and individualized, ethically sensitive care are essential in managing complex cases of severe anemia. Further studies are needed to better define the safety, efficacy, and optimal role of ESAs in this setting.
